# Evaluation of Surgical Options for Supernumerary Teeth in the Anterior Maxilla

**DOI:** 10.5005/jp-journals-10005-1529

**Published:** 2018-08-01

**Authors:** Marcello Maddalone, Elisa Rota, Ernesto Amosso, Gianluca Porcaro, Luca Mirabelli

**Affiliations:** 1 Deputy Chief, Department of Medicine and Surgery, University of Milano-Bicocca, Milan, Italy; 2 Specialist, Department of Medicine and Surgery, University of Milano-Bicocca, Milan, Italy; 3 House Officer, Department of Medicine and Surgery, University of Milano-Bicocca, Milan, Italy; 4 Adjunct Professor, Department of Medicine and Surgery, University of Milano-Bicocca, Milan, Italy; 5 House Officer, Department of Medicine and Surgery, University of Milano-Bicocca, Milan, Italy

**Keywords:** Anterior maxilla, Impacted teeth, Mesiodens, Supernumerary teeth, Surgical extraction.

## Abstract

**Aim:**

The purpose of this study was to evaluate the surgical options for supernumerary teeth (SNT) in the premaxillary region of children.

**Materials and methods:**

A total of 69 patients with 82 partially or completely formed SNT in the anterior maxillary region were identified over a 3-year period. All selected patients were assessed for the number, location, and family history of SNT, damage to adjacent roots, and associated symptoms. The surgical approaches used for removal were recorded. Postoperative clinical sequelae (loss of vitality, periodontal problems, pain, bleeding, and enanthema) were evaluated.

**Results:**

A total of 43, 30, and 9 SNT were extracted with palatal, buccal, and bicortical approaches respectively. Peri-odontal and vitality assessments revealed no problems in adjacent teeth at 6 months after surgery. Postoperative pain was within acceptable levels in all patients and subsided within 3 to 7 days. Mild postoperative bleeding occurred in eight patients. Enanthema subsided within 10 days in all patients.

**Conclusion:**

Our results suggest that early diagnosis and appropriate surgical treatment of SNT are important to decrease the risk of clinical complications.

**How to cite this article:** Maddalone M, Rota E, Amosso E, Porcaro G, Mirabelli L. Evaluation of Surgical Options for Supernumerary Teeth in the Anterior Maxilla. Int J Clin Pediatr Dent 2018;11(4):294-298.

## INTRODUCTION

Impacted SNT in the premaxillary region represent an interesting clinical entity that can have various influences on the dentition of neonates and present anatomical variations that frequently complicate surgery.

Supernumerary teeth are a relatively frequent odontogenic disorder^12^ and are defined as “teeth exceeding the usual number of 20 deciduous and 32 permanent teeth.” The most frequent clinical complications include the displacement or rotation of adjacent teeth before or after eruption, inhibition or delay of the eruption of normal adjacent teeth because of interference with available space, adjacent root resorption, crowding, malocclusion, dilaceration, delayed or abnormal permanent root development and, with anterior SNT, nasal cavity eruption and cyst and fistula formation.^1^

The incidence of mesiodens, defined as a SNT present in the midline between the two central incisors, in the permanent dentition of Caucasians is 0.15 to 3%^3,4^ with a higher incidence in East Asians. Furthermore, the incidence in men is twice that in women.

The SNT are most frequently located in the premaxilla and mandibular premolar region. In the primary dentition, they generally appear as normal-shaped or conical teeth, while in the permanent dentition, they may appear as conical, peg-shaped, or tuberculate teeth.^5-7^

Supernumerary teeth most commonly manifest as impacted maxillary anterior teeth, which can be divided into two general types according to the morphology: Supplemental SNT, characterized by dislocation, a normal or conical crown shape and a normal root size and shape; and rudimentary SNT, characterized by an abnormal shape and size (generally smaller) and a morphology similar to that of compound and complex odontomas.^8,9^

Clinical examination, followed by comprehensive radiological screening, is required for the accurate diagnosis and optimal management of SNT.

The management of SNT depends on their location, morphology, and presence or absence of clinical complications. For optimal surgical planning, computed tomography (CT) is normally mandatory for accurate identification of the location and appropriate selection of the surgical approach. The aim of this study was to evaluate the surgical options for SNT in the premaxillary region of children.

## MATERIALS AND METHODS

This study was approved by the Committee of Bioethics in Research of the S. Gerardo Hospital of Milano-Bicocca and patients gave their written consent to participate, where required.

From a pool of 2,159 patients who visited the surgical pedodontic service at the hospital for the first time over a period of 3 years, 69 patients with 82 partially or completely formed SNT in the anterior maxilla were selected for this study. The SNT were diagnosed through detailed clinical and radiological examinations performed by a single investigator. Orthopantomography and periapical radiographs were acquired for all patients. Computed tomography was performed when required by a radiologist with extensive experience in pediatric dentistry, including SNT diagnosis.

**Fig. 1:**
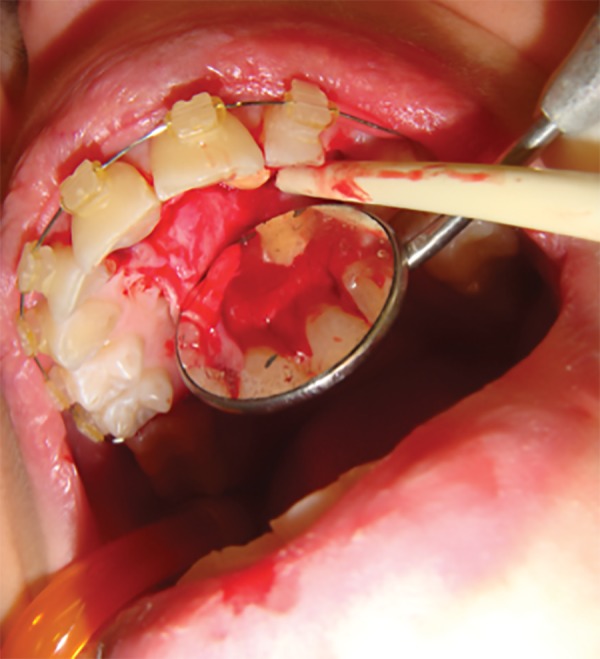
Full-thickness mucoDeriosteal flap

Patient data were collected from their charts and included age, gender, and reasons for visiting the hospital. All selected patients were assessed for the number of SNT, family history of SNT, adjacent root damage, location of SNT, and associated clinical symptoms. The surgical approaches used for removal, namely palatal, buccal, or bicortical were recorded. All surgical procedures, regardless of the approach used, included the elevation of a full-thickness, intrasulcular, mucoperiosteal flap (buccal, palatal, or both; Fig. 1). If SNT were not visible on flap elevation, a window was created in the bone wall for better access. When root separation was required for luxation of SNT, segmental resection using an air rotor handpiece was performed or the bicortical approach was adopted.

Postoperative clinical sequelae, including loss of vitality, periodontal problems, and discoloration in the adjacent teeth, pain, bleeding, and enanthema, were evaluated to identify the potential complications of surgery in this region.

All patients were monitored in the resting room after surgery until discharge and were asked to monitor and record episodes of bleeding at home. Four teeth on either side of the extracted tooth were evaluated for vitality, discoloration, and probing depths before and 1, 3, and 6 months after surgery. Postoperative pain was assessed using a visual analog scale (VAS) at the end of the procedure and the following day for all patients. If the pain persisted, it was assessed daily until it subsided. Postoperative enanthema formation and duration were also monitored and recorded for all patients.

## RESULTS

In total, there were 41 male and 28 female patients (1.46:1) with SNT, accounting for 3.19% of the total sample considered (2,159). The patient age ranged between 6 and 19 years, with an average age of 9 years and 8 months at the time of diagnosis. Multiple SNT were observed in the premaxilla of 11 patients, two of whom exhibited three SNT. One of these two patients was diagnosed with mucopolysaccharidosis.

Solitary SNT were the most common type (n = 51; 62.19%), followed by conical (n = 26; 31.70%) and rudimentary SNT (n = 5; 6.09%). Eighty SNT (97.56%) were permanent, while two (2.44%) were deciduous.

The primary reasons for visiting the hospital included routine radiological examination (n = 57; 69.52%), followed by permanent tooth displacement (n = 12; 14.63%), failure of permanent tooth eruption with or without clinical retention of primary teeth (n = 9; 10.97%) and SNT eruption (n = 4; 4.98%).

**Fig. 2:**
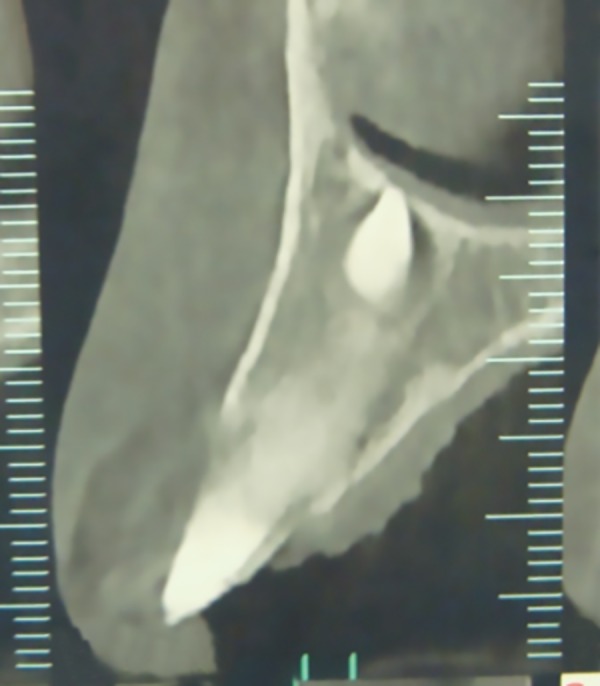
Distribution of SNT positions. Area 1: between the central incisors (mesiodens), n = 35 (42.68%). Area 2: lateral incisor region, n = 17 (20.74%). Area 3: canine region, n = 2 (2.44%). Area 4: superior to the roots of all teeth, n = 28 (34.14%)

We defined four anatomical regions in the premaxilla according to the relationship between SNT and the anatomical group of permanent teeth (Fig. 2):

- Area 1: Between the central incisors (mesiodens), n = 35 (42.68%)
- Area 2: Lateral incisor region, n = 17 (20.74%)
- Area 3: Canine region, n = 2 (2.44%)
- Area 4: Superior to the roots of all teeth, n = 28 (34.14%)

**Graph 1:**
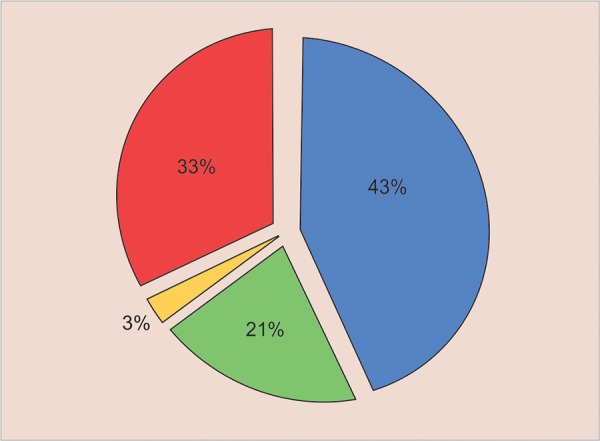
Position of a SNT oriented toward the nasal cavity

Thus, the region between the central incisors was the most common, followed by the region superior to the roots of all teeth. Among the 28 SNT found in the area immediately contiguous to the nasal region, 27 were either horizontal or directed toward the nasal cavity. The remaining one was inclined toward the occlusal plane. In patients aged up to 10 years, such rotated SNT made contact with the cortical bone at the base of the nasal cavity, with frequent areas of fenestration as observed on CT (Graph 1).

Among the remaining 54 SNT, 41 were vertical, nine mesially or distally inclined, and four horizontal. The four erupted SNT were completely developed, while 72 of the 78 unerupted ones showed completely developed crowns and incompletely developed roots. The remaining six showed an incomplete developed crown.

Of the 82 SNT, 63 (76.82%) were associated with a family history. The sagittal position of SNT was usually palatal in regions 1, 2, and 3 (41 of 54; 75 > 92%), while it was not very evident in region 4 (9 of 28; 32.14%). None of the SNT caused evident clinical damage to adjacent teeth, although several showed contact with the roots of adjacent teeth on radiographs. Therefore, a certain amount of subclinical damage to the cementum cannot be ruled out.

Disturbances in eruption or position of adjacent permanent teeth, including rotation, diastema or retarded eruption with or without retained primary teeth, were observed at the time of diagnosis in 38 patients.

Of the 82 SNT, 43, 30, and 9 were extracted via the buccal, palatal, and bicortical approaches respectively. All adjacent teeth were vital at 1, 3, and 6 months after surgery, with no discoloration or periodontal pocket formation. Postoperative pain was present in 26 patients, although it completely subsided within 3 days in 20 and within a week in six. The VAS score was generally low, not exceeding 6 (moderate) on a scale from 0 to 10. Postoperative bleeding occurred at home in eight patients, although it was easily controlled by compression by the parents. Only one patient required tranexamic acid for hemostasis. Various forms of enanthema developed in 36 patients, although it subsided within 10 days in all.

## DISCUSSION

Current data show that permanent SNT are observed in 0.8 to 3.2% of the general population,^10,11^ which is approximately equal to the prevalence of agenesis.

The SNT treatment is generally easier than agenesis treatment because it usually requires removal in the prepubertal age, as observed in several patients in our study. A delayed diagnosis can potentially lead to clinical problems, such as complex malpositioning or, eventually, root resorption.

Special attention should be given to newborns with a family history of SNT. In our study, 63 of 82 SNT (76.82%) were associated with a family history, indicating a strong genetic association.

In our country, routine dental examination during the first few years of age is performed by the pediatrician who periodically examines the child. A delay in eruption or rotation, particularly during the mixed dentition period, is the reason why most young individuals visit dental clinics, and it is at this time that symptomatic SNT are observed. The conjunction of the two events, i.e., the occurrence of SNT in relatives and a delay in eruption, strongly indicates the presence of SNT in children and adolescents. Asymptomatic SNT are usually detected on orthopantomograms obtained at the first orthodontic visit for treatment planning. Most children with a late diagnosis of SNT show better occlusion and are caries-free. However, a decreased prevalence of dental caries can be associated with an increased rate of complications associated with SNT because of late identification in the absence of dental x-ray examinations. The approximate time of diagnosis in our sample was the primary school-going age. Similar to that in other studies, the prevalence was higher in male patients, although this trait was less pronounced in our study.^12-14^ This was probably because the patients in our study did not belong to the general population; they included children referred by a general practitioner, orthodontist, or pediatric dentist. Another possible factor influencing the data was the inclusion of only the premaxillary area, not the entire dentition.

Our study showed that premaxillary SNT are generally solitary, concordant with the findings of other studies.^7,12,15-17^

Preceding papers evidenced care seeking reasons for diagnosis as we reported even if it usually did not focus attention on quantity of different casualties.^12,14,18^ The reason for the frequent detection of SNT on radiographs can be attributed to the fact that SNT are clinically asymptomatic in patients for many years. Furthermore, the paucity of erupted SNT in our study can be attributed to the fact that difficult cases were referred to our center by other dentists; general dentists generally extract uncomplicated SNT themselves and do not refer such cases. Interestingly, this could also be the reason for a large number of apically displaced SNT observed in our study. The removal of such SNT is particularly difficult and related to possible iatrogenic damage to the roots or neurovascular bundles of adjacent teeth; therefore, general dentists prefer to refer such cases to public care services. Original was in this type of SNT, the high prevalence of inverted vertical position has never been reported before.

The absence of serious clinical complications in our patients, unlike previous studies^3,10,18^ can be associated with early diagnosis in the majority of patients in our study, attributed to very efficient dental and medical services provided to school-going children in Monza. Furthermore, the deciduous maxillary anterior teeth are generally replaced early in the mixed dentition period; therefore, clinically symptomatic SNT can be detected in the early stages of development, before any damage to adjacent roots or periodontal structures can occur.

In total, 52 patients showed disturbances in eruption or position of adjacent permanent teeth at the time of diagnosis in our study, accounting for 96.29% SNT located between adjacent roots. Such SNT make significant contact with adjacent roots. Fortunately, there was no root damage caused by such SNT in our study, with the common complications including rotation (30-55.55%) or delayed eruption (22–0.74%). This supports the hypothesis that SNT usually cause dislocation of impacted permanent teeth, not root resorption, in the early stages of development.

## CONCLUSION

In this study, periodontal and vitality assessments revealed no permanent problems in the adjacent teeth after surgery. Furthermore, reversible signs and symptoms, including pain, bleeding, and enanthema, showed rapid resolution, with acceptable postoperative pain levels.

Early diagnosis of SNT can be facilitated in populations where basic dental surveillance is provided for young individuals. Trained dentists or pediatricians can screen cases with clinical symptoms indicating the presence of SNT, using basic radiological examination, such as digital orthopantomography or cone beam CT to confirm the diagnosis. Digital radiography requires low patient exposure; therefore, it should be used for the screening of young school-going children with a family history of SNT, even if they show normal tooth positions and craniofacial relationships. This can prevent clinical complications and significant clinical damage, such as root resorption. Early intervention also prevents tooth dislocation and the requirement for future orthodontic treatment to correct malocclusion.
